# Best Treatment Option for Patients With Refractory Aggressive B-Cell Lymphoma in the CAR-T Cell Era: Real-World Evidence From GELTAMO/GETH Spanish Groups

**DOI:** 10.3389/fimmu.2022.855730

**Published:** 2022-07-12

**Authors:** Mariana Bastos-Oreiro, Antonio Gutierrez, Juan Luís Reguera, Gloria Iacoboni, Lucía López-Corral, María José Terol, Valentín Ortíz-Maldonado, Jaime Sanz, Luisa Guerra-Dominguez, Rebeca Bailen, Alberto Mussetti, Pau Abrisqueta, Rafael Hernani, Hugo Luzardo, Juan-Manuel Sancho, Javier Delgado-Serrano, Antonio Salar, Carlos Grande, Leyre Bento, Sonia González de Villambrosía, Daniel García-Belmonte, Anna Sureda, Antonio Pérez-Martínez, Pere Barba, Mi Kwon, Alejandro Martín García-Sancho

**Affiliations:** ^1^ Hospita Universitario Gregorio Marañón, Instituto de Investigación Sanitaria Gregorio Marañón, Madrid, Spain; ^2^ Hospital Universitario Son Espases, Fundació Institut d'Investigació Sanitària Illes Balears (IdISBa), Palma de Mallorca, Spain; ^3^ Hematology Department, Hospital Universitario Virgen del Rocío, Sevilla, Spain; ^4^ Hematology Department, Hospital Vall d’ Hebron, Barcelona, Spain; ^5^ Hospital Universitario de Salamanca, Instituto de investigación biomédica de Salamanca (IDBAL), CIBERONC, Salamanca, Spain; ^6^ Hematology Department, Hospital Clínico Universitario de Valencia, Valencia, Spain; ^7^ Hematology Department, Hospital Clinico Universitario de Barcelona, Barcelona, Spain; ^8^ Hematology Department, Hospital Universitario La Fé de Valencia, Valencia, Spain; ^9^ Hospital Negrin, Las Palmas de Gran Canari, Las Palmas de Gran Canarias, Spain; ^10^ Institut Català d’Oncologia-Hospitalet, Instituto de Investigación Biomédica de Bellvitge (IDIBELL), Universitat de Barcelona, Barcelona, Spain; ^11^ Hematology Department, Campus ICO-Germans Trias i Pujol (ICO-IJC)-Hospital Germans Trias i Pujol, Badalona, Spain; ^12^ Hematology Department, Hospital del Mar, Barcelona, Spain; ^13^ Hematology Department, Hospital 12 de Octubre, Madrid, Spain; ^14^ Hematology Department, Hospital Universitario de Marques de Valdecillas, Santander, Spain; ^15^ Hematology Department, Hospital Universitario de La Zarzuela, Madrid, Spain; ^16^ Hematology Department, Hospital Universitario La Paz, Madrid, Spain

**Keywords:** refractory aggressive B cell lymphoma, CAR-T cell therapy, standard of care (SOC), real world evidence (RWE), scholar-1 criteria

## Abstract

Real-world evidence comparing the efficacy of chimeric antigen receptor (CAR) T-cell therapy against that of the previous standard of care (SOC) for refractory large B-cell lymphoma (LBCL) is scarce. We retrospectively collected data from patients with LBCL according to SCHOLAR-1 criteria treated with commercial CAR T-cell therapy in Spain (204 patients included and 192 treated, 101 with axicabtagene ciloleucel [axi-cel], and 91 with tisagenlecleucel [tisa-cel]) and compared the results with a historical refractory population of patients (n = 81) obtained from the GELTAMO-IPI study. We observed superior efficacy for CAR-T therapy (for both axi-cel and tisa-cel) over pSOC, with longer progression-free survival (PFS) (median of 5.6 vs. 4–6 months, p ≤ 0.001) and overall survival (OS) (median of 15 vs. 8 months, p < 0.001), independently of other prognostic factors (HR: 0.59 (95% CI: 0.44–0.80); p < 0.001] for PFS, and 0.45 [(95% CI: 0.31–0.64)] for OS). Within the CAR-T cohort, axi-cel showed longer PFS (median of 7.3 versus 2.8 months, respectively, p = 0.027) and OS (58% versus 42% at 12 months, respectively, p = 0.048) than tisa-cel. These differences were maintained in the multivariable analysis. On the other hand, axi-cel was independently associated with a higher risk of severe cytokine release syndrome and neurotoxicity. Our results suggest that the efficacy of CAR-T cell therapy is superior to pSOC in the real-world setting. Furthermore, axi-cel could be superior in efficacy to tisa-cel, although more toxic, in this group of refractory patients according to SCHOLAR-1 criteria.

## Introduction

Diffuse large B-cell lymphoma (DLBCL) is the most common non-Hodgkin’s lymphoma (NHL) (1, 2). The incorporation of the monoclonal antibody rituximab into first-line treatment regimens has led to improved survival of patients with DLBCL (3). However, approximately 40% of such patients will present refractory or relapsed (r/r) disease after the first-line treatment (4). Classically, the prognosis of patients with r/r DLBCL is poor, considering that salvage treatment followed by high-dose chemotherapy and autologous stem cell transplantation (auto-SCT) will be a curative option for only 20%–25% of patients (5–8).

The prognosis is especially poor for refractory patients, as was highlighted in the SCHOLAR-1 study, the largest retrospective analysis of this patient population (9). This study pooled data from two separate phase III clinical trials (the Lymphoma Academic Research Organization-CORAL and the Canadian Cancer Trials Group LY.12) and two observational cohorts (MD Anderson Cancer Center and the University of Iowa/Mayo Clinic Lymphoma Specialized Program of Research Excellence). Patients with DLBCL refractory to first-line or subsequent lines of therapy, or relapsing within 1 year after auto-SCT, had a very low chance of responding to the next line of treatment (26% overall response rate [ORR] and 7% complete response [CR]) and a median overall survival (OS) of only 6.3 months (9). These findings underscored the considerable unmet need for effective therapies for patients with refractory DLBCL and have served as benchmarks for assessing novel therapies in this patient population (10).

Chimeric antigen receptor (CAR) T-cell therapy constitutes a paradigm shift in the treatment of r/r DLBCL (11). Currently, two CAR T‐cell products targeting CD19 have been approved in Europe and three in the United States for treating r/r DLBCL after at least two lines of systemic therapy: axicabtagene ciloleucel (axi‐cel) (12, 13), tisagenlecleucel (tisa-cel) (14), and lisocabtagene maraleucel (liso-cel) (15) (16). The three pivotal single-arm phase II clinical trials evidenced highly encouraging results, showing complete response rates of 40%–58% and prolonged remission in 30%–40% of patients (12–16). These pivotal trials had important differences in the inclusion criteria as well as the study designs. In this sense, while the ZUMA-1 trial (axi-cel) included only refractory patients according to the SCHOLAR-1 criteria, the JULIET (tisa-cel) and TRANSFORM (liso-cel) trials included also non-refractory patients.

Real-world data on CAR-T therapy from various countries in Europe and the United States have shown similar efficacy to those of the pivotal trials (17–21). To date, there has been little evidence comparing CAR T-cell therapy in real world versus the previous standard of care (pSOC) of the pre-CAR era (17, 22). Moreover, none of the real-world evidence (RWE) studies have focused on analyzing refractory patients, the population of DLBCL with the highest unmet need.

In the present study, we aim to describe the global Spanish experience with CAR T-cell therapy in the commercial setting and compare these results with the historical treatment prior to the CAR T-cell therapy, focusing our analysis on refractory patients according to the Scholar-1 criteria.

## Methods

### Study Design and Patients

We performed a multicenter, retrospective, observational study conducted in accordance with the Declaration of Helsinki and approved by the institutional ethics committee of Hospital Universitario Gregorio Marañón.

We included in the CAR-T cohort all adult patients treated with commercially available CAR-T cell products with aggressive B-cell lymphoma different from primary mediastinal large B-cell lymphoma (PMLBCL) who were registered in the GELTAMO/GETH-TC (Grupo Español de Linfomas y Trasplante Autólogo de Médula Ósea/Grupo Español de Trasplante Hematopoyético y Terapia Celular) database of patients treated with CAR T-cell therapy in eight Spanish centers between February 2019 and July 2021. All patients were judged eligible by the Expert Committee of the Spanish National Health System. Patients fulfilling SCHOLAR-1 criteria (9) for refractory disease (progressive disease as best response to any line of therapy, stable disease as best response to ≥4 cycles of first-line therapy or ≥2 cycles of later-line therapy, or relapse <12 months after auto-SCT) were identified and included in the primary analysis. Patient selection, supportive care, toxicity management, and response assessment followed institutional practices. Cytokine release syndrome (CRS) and neurotoxicity were graded according to the according to the ASTCT consensus criteria (23, 24).

This cohort was compared with a historical population of r/r DLBCL patients from the GELTAMO-IPI study, treated in the pre-CAR-T-cell therapy era (pSOC) (25). The GELTAMO-IPI study included patients from 20 academic and community hospitals in the GELTAMO network in Spain, diagnosed between 1998 and 2014, and treated with R-CHOP or more intense regimens with a curative intent. For the present study, centers were required to update the follow-up and additional data about the treatment at relapse or progression. Finally, nine centers (n = 9) agreed to participate in the present study. As previously mentioned for the CAR-T cohort, only patients who met at least one of the Sholar-1 refractory criteria were considered for this cohort as well.

### Statistical Analysis

The present analysis was based on a data cutoff on July 25, 2021, and March 1, 2019, for the CAR-T and pSOC cohorts, respectively. Descriptive statistics, including median, and interquartile range (IQR) for the continuous variables and percentages for the categorical variables, were obtained. To evaluate the association between two categorical variables, we employed Fisher’s exact test or the chi-squared test.

The median follow-up time (in months) was calculated for the surviving patients by the reverse Kaplan–Meier method. The time to event, overall survival (OS), and progression-free survival (PFS) were estimated using the Kaplan–Meier method, and comparisons between variables of interest were performed using the log-rank test. The OS for the ITT and infused populations was calculated from start of treatment for the pSOC cohort and apheresis and infusion date, respectively, until the date of death from any cause, censoring for patients alive at last contact. The PFS for the ITT and infused populations was calculated from failure to last treatment and infusion date, respectively, until the date of relapse, progression, or death from any cause, censoring for patients alive and progression-free at last contact. For the comparison analysis between the pSOC and ITT CAR-T cohorts, the survival was measured from the failure to last treatment in the patients fulfilling the Scholar-1 criteria. This analysis was exploratory, and p-values were not corrected for multiple testing. The specific cutoffs for several quantitative variables such as time to approval, apheresis or infusion, ferritin, and C-reactive protein (CRP) were calculated using receiver operating characteristic curves.

To assess the effect of important covariates on response and toxicity, we performed a multivariable logistic regression. We also performed a multivariate survival analysis with the variables that appeared to be significant in the univariate analysis (p < 0.05), according to the Cox proportional hazard regression model. All reported p-values were two-sided, and statistical significance was defined at p < 0.05. Analyses were performed using SPSS version 25 (SPSS, Chicago, IL).

## Results

### CAR-T Cohort Characteristics

From the initial population of 255 patients registered in the GELTAMO/GETH-TC database, 204 patients meeting the SCHOLAR-1 criteria for refractory disease were included in the intention-to-treat (ITT) analysis. Fifty-one patients were excluded due to non-refractory disease (15 patients), presence of PMLBCL histology (19 patients), histology different from aggressive B-cell lymphoma (4 patients), or lack of data or follow-up (13 patients) (**Supplementary Figure 1**).

Patient characteristics are shown in **Table 1**. Of the 204 refractory patients included in the ITT analysis, 192 (94%) were infused. The median time from official approval to infusion was 60 days (IQR: 49–73 days), and the median time from apheresis to infusion was 46.5 days (IQR: 39–55.7 days). For the infused patients, 101 were administered axi-cel and 91 were administered tisa-cel. Regarding the SCHOLAR-1 criteria in the ITT analysis, 64% of the patients were primary refractory, 82% were refractory to the last therapy, and 27% had an early relapse after auto-SCT. The most common histology included was DLBCL, not otherwise specified (NOS) (77%), followed by transformed follicular lymphoma (13%), high-grade B-cell lymphoma double or triple hit (9%), and high-grade B-cell lymphoma, NOS (1%). Seventeen patients (9%) had an Eastern Cooperative Oncology Group performance status (ECOG-PS) ≥2 pre-lymphodepletion, and 11 (5%) pre-apheresis. The revised IPI (R-IPI) was high (3–5) at diagnosis in 55% of the patients and pre-lymphodepletion in 54% of the patients. Bulky disease (more than 10 cm for the larger diameter) was present in 37% of the patients pre-apheresis. Almost one-third (29%) of the patients previously underwent auto-SCT. Eighty-two percent of the patients required bridging therapy. The most common bridging therapies were gemcitabine-based regimens (35%), cyclophosphamide-based regimens (24%), intense salvage regimens (R-ICE [rituximab plus ifosfamide, carboplatin, and etoposide], R-DHAP [rituximab plus dexamethasone, ara-C, and cisplatin], MINE [mesna, ifosfamide, mitoxantrone, and etoposide]) (13%), bendamustine-based regimens (10%), radiotherapy (5%), steroid monotherapy (5%), lenalidomide-based regimens (2%), nivolumab (1%), and brentuximab (1%).

**Table 1:** Comparison of the previous standard-of-care (pSOC) cohort versus CAR-T cell, intention to treat (ITT), and infused cohorts.

	pSOC cohort	CAR-T cell cohort	*p*	CAR-T cell cohort	*p*
	(N = 81)	(N = 198)		(N = 192)	
**Median age** (IQR):	62 (49–74)	55 (48–64)	**<0.001**	55 (47–64)	**<0.001**
**Gender** (M/F) (%):	61%/39%	64%/36%	0.78	63%/37%	0.89
**Diagnosis**:			0.09		0.1
** -** DLBCL NOS	68 (84%)	156 (79%)		151 (79%)	
** -** TFL	3 (4%)	23 (12%)		22 (11%)	
** -** HGL DH/TH	7 (9%)	17 (9%)		17 (9%)	
** -** HGL NOS	3 (4%)	2 (1%)		2 (1%)	
**Ann Arbor stage,** n (%):			0.86		0.86
** -** I–II	14 (17%)	32 (16%)		32 (17%)	
** -** III–IV	66 (82%)	165 (84%)		159 (83%)	
**R-IPI score,** n (%):			0.58		0.58
** -** Favorable (0–2)	32 (42%)	78 (46%)		76 (46%)	
** -** Unfavorable (3–5)	45 (58%)	93 (54%)		90 (54%)	
**Previous ASCT,** n (%):	27 (34%)	58 (29%)	0.47	58 (30%)	0.57
**Median previous lines** (IQR):	2 (1–2)	2 (2–3)	**<0.001**	2 (2–3)	**<0.001**
**More than 2 previous lines,** n (%):	17 (21%)	84 (42%)	**<0.001**	79 (41%)	**0.001**
**Bulky disease,** n (%):	29 (37%)	68 (35%)	0.78	66 (35%)	0.78
**Median follow-up** (95% CI):	93 (82–105)	11 (9–14)	**<0.001**	11 (9–14)	**<0.001**
**Median PFS** (95% CI):	4.6 (1.5–7.7)	5.1 (3.5–6.7)	**0.004**	5.6 (3.7–7.5)	**0.002**
**12-month PFS** (95% CI):	22% (18–27)	36% (32–39)	**0.004**	37% (33–40)	**0.002**
**Median OS** (95% CI):	8.2 (6.6–9.9)	14.5 (NA)	**<0.001**	15 (NA)	**<0.001**
**12-month OS** (95% CI):	36% (31–42)	54% (50–58)	**<0.001**	55% (51–60)	**<0.001**

IQR, interquartile range; ASCT, autologous stem cell transplantation; DLBCL, NOS, diffuse large B-cell lymphoma not otherwise specified; HGL DH/TH, high-grade B-cell lymphoma double and triple hit; HGL, NOS, high-grade B-cell lymphoma not otherwise specified; ITT, intention to treat; TFL, transformed follicular lymphoma: transformed; OS, overall survival; PFS, progression-free survival; pSOC, previous standard of care; R-IPI, revised international prognostic index.

Results with statistical significance have been highlighted in bold.

### Standard-of-Care Cohort Characteristics

As shown in **Table 1**, characteristics of the pSOC cohort were similar to those of the CAR-T cohort, except for age, which was significantly higher in the pSOC cohort. Similar to the CAR-T cohort, the most common histology included was DLBCL, NOS (84%). The R-IPI was high (3–5) at diagnosis in 58% of the patients. All patients received at least two lines of therapy, with 21% receiving more than three lines previous to the last therapy. Regarding the SCHOLAR-1 criteria, 50% of patients were primary refractory, 71% were refractory to the last therapy, and 17% had an early relapse after auto-SCT. The median number of lines was 2 (IQR: 1–2). The last therapies received were R-GemOx (19%), R-bendamustine (15%), R-ICE (13%), R-ESHAP (etoposide, methyl prednisolone, cytosine arabinoside, cisplatinum) (10%), mini-BEAM (BCNU, etoposide, cytosine arabinoside, melphalan) (5%), R-GIFOX (rituximab plus gemcitabine, ifosfamide, and oxaliplatin) (4%), lenalidomide (4%), and others (30%). One-third of the patients underwent hematopoietic auto-SCT (34%), and 8.6% underwent allogeneic transplantation.

### CAR-T Versus pSOC

**Figure 1:**
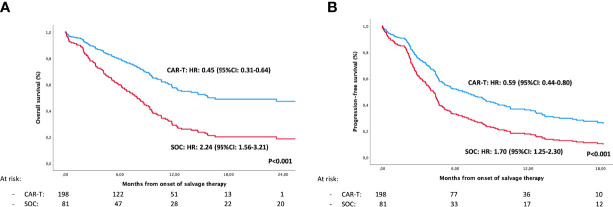
Intention to treat analysis of overall survival **(A)** and progression-free survival **(B)** in previous standard of care (pSOC) versus CAR-T cell cohorts, adjusted using a multivariable Cox regression model.

**Figure 2:**
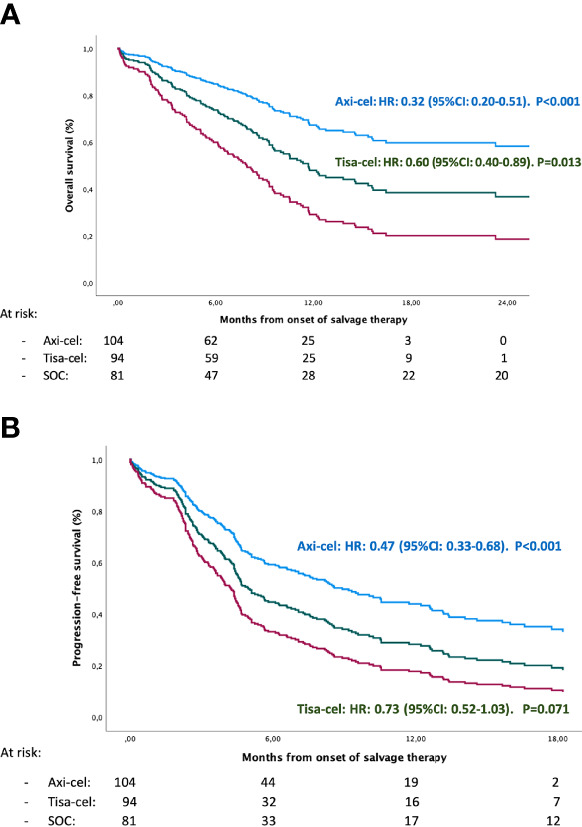
Overall survival **(A)** and progression-free survival **(B)** comparing: Axi-cel vs Tisa-cel vs previous Standardregression of care (pSOC) adjusted using a multivariable Cox regression model.

The median follow-up was significantly longer for the pSOC cohort than for the CAR-T cohort (**Table 1**), 93 months vs. 11 months since start of treatment and apheresis, respectively. Refractory patients treated with CAR T-cells versus pSOC had significantly longer PFS (median of 5.1 and 5.6 vs. 4.6 months, p ≤ 0.001) and better OS (median of 14.5 and 15 vs. 8.2 months, p < 0.001), considering both the ITT and infused populations (**Table 1**; **Figure 1**). When analyzing separately by the various products versus pSOC, the differences were in favor of both products over the pSOC (**Table 2**; **Figure 2** **)**, with a median PFS of 4.6 months (95% CI 2.9–8.5) for the pSOC cohort versus 8.5 months (95% CI 6.6–14.4) (p < 0.001) for axi-cel and 4.6 months (95% CI 4.3–8) (p = 0.074) for tisa-cel, and median OS of 8.2 months (95% CI 6.6–9.9) for the pSOC cohort versus not reached (p < 0.001) for axi-cel and 11.7 months (95% CI 7.8–15.6) (p = 0.015) for tisa-cel.

**Table 2:** Comparison of the previous standard-of-care (pSOC) cohort and infused CAR-T cell, axi-cell, and tisa-cel cohorts.

	pSOC cohort (N=81)	Axi-cel cohort (n=101)	*p*	Tisa-cel cohort (n=91)	*p*
**Median age** (IQR)	62 (49–74)	54 (44–62)	**<0.001**	56 (50–65)	**0.012**
**Gender** (M/F) (%)	61%/39%	60%/40%	1	65%/35%	0.64
**Diagnosis**: - HGL DH/TH - HGL NOS - DLBCL NOS - tFollicular	7 (9%) 3 (4%) 68 (84%) 3 (4%)	6 (6%) 1 (1%) 83 (82%) 11 (11%)	0.17	11 (12%) 1 (1%) 68 (75%) 11 (12%)	0.12
**Ann Arbor stage**: - I–II - III–IV	14 (17%) 66 (82%)	16 (16%) 84 (84%)	0.84	16 (18%) 75 (82%)	1
**R-IPI score**: - 0–2 - 3–5	32 (42%) 45 (58%)	41 (47%) 47 (53%)	0.53	35 (45%) 43 (55%)	0.75
**ASCT** (%):	27 (34%)	28 (28%)	0.42	30 (33%)	1
**Median previous lines** (IQR):	2 (1–2)	2 (2–3)	**<0.001**	2 (2–3)	**<0.001**
**More than 2 previous lines**, n (%):	17 (21%)	36 (36%)	**0.034**	43 (47%)	**<0.001**
**Bulky disease**, n (%):	29 (37%)	34 (34%)	0.64	32 (36%)	0.87
**Median follow-up** (95% CI):	93 (82–105)	10 (8–12)	**<0.001**	14 (10–18)	**<0.001**
**Median PFS** (95% CI):	4.6 (1.5–7.7)	8.5 (2.8–14.2)	**<0.001**	4.6 (4.1–5.2)	**0.074**
**12-month-PFS** (95% CI):	22% (18–27)	46% (40–51)	**<0.001**	28% (23–33)	**0.074**
**Median OS** (95% CI):	8.2 (6.6–9.9)	NR	**<0.001**	11.7 (7.8–15.6)	**0.015**
**12-month-OS** (95% CI):	36% (31–42)	61% (55–67)	**<0.001**	49% (43–55)	**0.015**

IQR, interquartile range; ASCT, autologous stem cell transplantation; DLBCL, NOS, diffuse large B-cell lymphoma not otherwise specified; HGL DH/TH, high-grade B-cell lymphoma double and triple hit; HGL, NOS, high-grade B-cell lymphoma not otherwise specified; TFL, transformed follicular lymphoma: transformed; OS, overall survival; PFS, progression-free survival; pSOC, previous standard of care; R-IPI, revised international prognostic index.

Results with statistical significance have been highlighted in bold.

To account for possible imbalances between the groups, we performed a multivariate analysis using Cox regression, including all the patients from both cohorts. We found that pSOC therapy, as well as not having undergone auto-SCT, had a significant adverse impact on PFS and OS (**Supplementary Table 1**).

### CAR T-Cell Cohort Efficacy and Toxicity

**Table 3** shows the characteristics of the CAR-T cell cohort at diagnosis and at CAR T-cell therapy. The responses to CAR T-cell therapy are summarized in **Supplementary Table 2**. The best response rates after CAR T-cell infusion in this refractory group of patients meeting the Scholar-1 criteria (n = 192) were 36% for complete response, 24% for partial response, 7% for stable disease, and 22% for progressive disease (**Supplementary Table 2**).

**Table 3:** CAR-T cell cohort characteristics: intention-to-treat and infused axi-cel and tisa-cel populations.

	CAR-T cell cohort. ITT	CAR-T cell	CAR-T cell	p
	(N = 198)	Axi-cel infused	Tisa-cell infused	
		(N = 101)	(N = 91)	
**At diagnosis**				
Median age (IQR):	55 (48–64)	54 (44–62)	56 (50–65)	**0.058**
Gender (M/F) (%):	64%/36%	60%/40%	65%/35%	0.55
Diagnosis:				0.48
- DLBCL NOS	156 (79%)	83 (82%)	68 (75%)	
- tFollicular	23 (12%)	11 (11%)	11 (12%)	
- HGL DH/TH	17 (9%)	6 (6%)	11 (12%)	
- HGL NOS	2 (1%)	1 (1%)	1 (1%)	
			
Ann Arbor stage:				0.85
- III–IV	165 (84%)	84 (84%)	75 (82%)
R-IPI score:				0.88
- Favorable (0–2)	78 (46%)	41 (47%)	35 (45%)
- Unfavorable (3–5)	93 (54%)	47 (53%)	43 (55%)
**Previous therapy**				
ASCT (%):	58 (29%)	28 (28%)	30 (33%)	0.44
Median previous lines (IQR):	2 (2-3)	2 (2-3)	2 (2-3)	0.28
**Pre-apheresis**				
Median age (IQR):	59 (50-67)	57 (47-65)	61 (52-68)	0.097
Primary refractory:	128 (65%)	65 (64%)	58 (64%)	1
Refractory to previous line:	163 (82%)	82 (81%)	75 (82%)	0.85
Status pre-apheresis:				0.069
- CR	2 (1%)	0 (0%)	2 (2%)
- PR	9 (4%)	4 (4%)	5 (5%)
- SD	24 (12%)	8 (8%)	16 (18%)
- Progression or relapse	163 (82%)	89 (88%)	68 (75%)
ECOG **>**1:	9 (5%)	2 (2%)	5 (6%)	0.25
Ann Arbor stage:				0.16
- I–II	40 (20%)	17 (17%)	23 (26%)
- III–IV	155 (80%)	83 (83%)	66 (74%)
Bulky disease (≥10 cm):	73 (37%)	46 (45%)	25 (28%)	**0.016**
**Pre-lymphodepletion**				
Disease status:				0.4
- CR	7 (4%)	4 (4%)	3 (3%)
- PR	17 (9%)	12 (12%)	5 (6%)
- SD	51 (27%)	24 (25%)	27 (31%)
- Progression	114 (60%)	57 (59%)	53 (60%)
ECOG-PS **>**1:	17 (8%)	9 (9%)	3 (3%)	0.14
Ann Arbor stage:				0.21
- I–II	27 (16%)	11 (12%)	16 (20%)
- III–IV	146 (84%)	79 (88%)	64 (80%)
Bulky disease (≥10 cm):	80 (42%)	53 (53%)	26 (29%)	**<0.001**
R-IPI score:				0.3
- Favorable (0–2)	85 (46%)	40 (42%)	43 (50%)
- Unfavorable (3–5)	99 (54%)	55 (58%)	43 (50%)

IQR, interquartile range; ASCT, autologous stem cell transplantation; DLBCL, NOS, diffuse large B cell lymphoma not otherwise specified; HGL DH/TH, high-grade B-cell lymphoma double and triple hit; HGL, NOS, high-grade B-cell lymphoma not otherwise specified; ITT, intention to treat; TFL, transformed follicular lymphoma: transformed; OS, overall survival; PFS, progression-free survival; pSOC, previous standard of care; R-IPI, revised international prognostic index.

Results with statistical significance have been highlighted in bold.

For all infused patients (n = 192), the median follow-up from infusion was 10 months (95% CI: 8–12 months), the median PFS was 3.3 months (95% CI 2–4.6), and the median OS was 11.8 months; the estimated 12-month PFS and OS were 35% (95% CI 27–42) and 50% (95% CI 41–59), respectively, and the 12-month PFS and OS for the patients who achieved complete response were 73% (95% CI 61–85) and 86% (95% CI 76–95), respectively. The factors affecting PFS and OS in the univariate analysis are shown in **Table 4**. In the multivariable analysis, the factors with independent adverse influence on PFS were CAR-T cell type (tisa-cel), unfavorable R-IPI at lymphodepletion, non-previous ASCT, and HCTCI (Hematopoietic Cell Transplantation-specific Comorbidity Index) 3–7 pre-lymphodepletion, and those for OS were CAR-T cell type (tisa-cel), unfavorable R-IPI at lymphodepletion, ECOG-PS 2–4 pre-apheresis, primary refractory disease, and HCTCI 3-7 pre-lymphodepletion (**Table 5**).

**Table 4:** Univariable analysis of survival in CAR-T-infused patients.

	12m-OS (95% CI)	p	12m-PFS (95% CI)	p

**AT DIAGNOSIS**				
Age:		0.39		0.17
	52% (40–63)		38% (28–47)	
**-** 18–60 years	46% (31–62)		29% (17–42)	
		
**-** >60 years				
Sex:		0.68		0.65
	46% (35–58)		33% (24–43)	
**-** Male	56% (41–71)		38% (25–50)	
		
**-** Female				
Diagnosis:		0.37		0.2
	46% (36–57)		33% (24–41)	
**-** DLBCL NOS	78% (59–97)		50% (22–78)	
	50% (21–79)		35% (13–58)	
**-** Follicular transformed	0% (NA)		0% (NA)	
		
**-** HGL DH/TH				
		
**-** HGL NOS				
Ann Arbor stage:		0.74		0.54
	48% (26–70)		29% (11–47)	
**-** I–II	50% (40–60)		36% (27–44)	
		
**-** III–IV				
R-IPI:		0.57		0.95
**-** Favorable (0–2)	50% (36–65)		35% (24–47)	
	46% (32–60)		32% (20–44)	
**-** Unfavorable (3–5)				
PREVIOUS THERAPY				
Previous ASCT:		**0.026**		**0.039**
	62% (47–77)		46% (32–60)	
**-** Yes	43 (31–55)		30% (20–39)	
		
**-** No				
Number of previous lines:		0.29		**0.27**
**-** 0–2	51% (39–64)		37% (26–47)	
**-** >2	47% (34–61)		32% (21–43)	
**STATUS PRE-APHERESIS**				
Primary refractory:		**0.02**		0.27
	42% (31–54)		33% (23–42)	
**-** Yes	63% (49–78)		39% (26–52)	
		
**-** No				
Refractory to previous line:		**0.02**		**0.015**
	45% (35–55)		31% (22–39)	
**-** Yes	70% (53–88)		53% (34–72)	
		
**-** No				
Status pre-AF:		0.35		0.5
	100% (NA)		100% (NA)	
**-** CR	75% (45–100)		67% (36–97)	
	57% (34–81)		29% (9.8–48)	
**-** PR	46% (35–56)		33% (24–41)	
		
**-** SD				
		
**-** Progression				
ECOG-PS preAF:		**<0.001**		**0.014**
	51% (41–60)		36% (28–43)	
**-** 0–1	0 (NA)		0 (NA)	
		
**-** 2–4				
Ann Arbor stage pre-apheresis:		0.42		0.56
**-** I–II	40% (21–59)		26% (9–43)	
**-** III–IV	52% (41–62)		36% (27–45)	
		
		
Bulky pre-apheresis (≥10 cm):		0.59		0.36
**-** Yes	50% (39–62)		34% (22–46)	
**-** No	50% (35–65)		36% (26–46)	
		
		
**CAR-T DATA**				
Type of CAR-T:		**0.048**		**0.023**
**-** Axi-cel	58% (45–71)		40% (28–52)	
**-** Tisa-cel	42% (29–54)		28% (19–38)	
		
		
Bridging therapy:		**0.038**		**0.012**
	45% (35–55)		30% (22–39)	
**-** Yes	72% (51–92)		56% (38–74)	
		
**-** No				
**STATUS AT CART**				
**Disease status:**		0.082		**0.007**
**-** Progression/stable disease	46% (46–56)		30% (22–37)	
**-** Partial response	67% (35–98)		69% (42–96)	
**-** Complete response	100% (NA)		75% (32–100)	
**HCTCI:**		**<0.001**		**0.004**
**-** 0–2	59% (48–70)		42 (32–51)	
**-** 3–7	21% (7–35)		18 (5–31)	
**ECOG-PS:**		0.35		0.16
**-** 0–1	50% (41–60)		35% (27–43)	
**-** 2–4	48% (15–80)		22% (0–48)	
**Ann Arbor** stage:		0.34		0.37
**-** I–II	48% (23–74)		38% (16–60)	
**-** III–IV	50% (39–60)		34% (26–43)	
**Bulky mass** (≥10 cm):		0.53		0.41
**-** Yes	52% (39–66)		33% (21–44)	
**-** No	47% (35–59)		36% (25–46)	
**R-IPI:**		**0.013**		**0.006**
**-** Favorable (0–2)	60% (47–74)		43% (32–55)	
**-** Unfavorable (3–5)	42% (29–54)		27% (17–38)	

DLBCL NOS, diffuse large B-cell lymphoma not otherwise specified; tFollicular, transformed; PML, primary mediastinal lymphoma; HGL DH/TH, high-grade lymphoma double and triple hit; HGL NOS, high-grade lymphoma not otherwise specified; HCTCI, hematopoietic cell transplantation-specific comorbidity index; PS, performant status; ASCT, autologous stem cell transplantation; R-IPI, reviewed international prognostic index; PFS, progression-free survival; OS, overall survival.

Results with statistical significance have been highlighted in bold.

**Table 5:** Multivariable analysis of survival in CAR-T-infused patients.

	HR	p value	95% CI
**For PFS**			
**CAR-T type (tisa-cel)**	1.74	0.009	1.15–2.63
**R-IPI unfavorable (3–5) pre-lymphodepletion**	1.76	0.007	1.17–2.64
**HCTCI pre-lymphodepletion 3–7**	2.15	<0.001	1.38–3.35
**Non previous ASCT**	1.66	0.036	1.03–2.65
**For OS**			
**CAR-T type (tisa-cel)**	1.96	0.011	1.17–2.28
**Primary refractory**	2.07	0.015	1.15–3.73
**ECOG PS 2–4 pre-apheresis**	3.17	0.028	1.03–9.74
**HCTCI pre-lymphodepletion 3–7**	3.62	<0.001	2.14–6.12
**R-IPI unfavorable (3–5) pre-lymphodepletion**	1.7	0.047	1.01–2.86

PFS, progression-free survival; OS, overall survival; ASCT, autologous stem cell transplantation; HCTCI, hematopoietic cell transplantation-comorbidity index; R-IPI, reviewed international prognostic index; HR, hazard ratio; CI, confidence interval.

**Figure 3:**
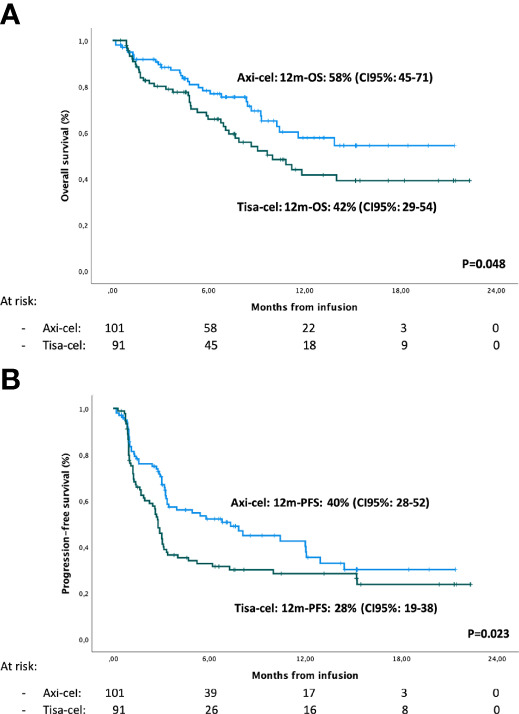
Overall survival **(A)** and progression-free survival **(B)** in axi-cel and tisa-cel cohorts.

For the infused patients (n = 192), axi-cel was superior to tisa-cel for PFS and OS, with a median PFS of 7.3 (95% CI 3.3–11.3) versus 2.8 months (95% CI 2.4–3.1) (p = 0.027), and median OS not reached (NR) versus 10 months (95% CI 6.6–13.4) (p = 0.048), respectively (**Figure 3**). Similarly, the 12-month PFS was 40% (95% CI 28–52) vs. 28% (95% CI 19–38) (p = 0.023), and the 12-month OS was 58% (95% CI 45–71) and 42% (95% CI 29–54) (p = 0.048) for axi-cel and tisa-cel cohorts, respectively (**Tables 4**, **5**).

A total of 78% of patients experienced some degree of CRS, with only 6% of cases grade 3 or more. The incidence of CRS was significantly higher in the patients treated with axi-cel than those treated with tisa-cel (90% vs. 65%, p < 0.001); however, the incidence of severe CRS did not differ between the two CAR-T cohorts (8% vs. 4%, p = 0.38). In terms of neurotoxicity, 29% of the patients had some degree of neurotoxicity, with 11% experiencing grade 3 or more. These events were more frequent with axi-cel than with tisa-cel: 42 (42%) vs. 13 (14%) patients, respectively (p < 0.001), and severe neurotoxicity occurred in 16 (16%) and 5 (5%) patients (p = 0.022), respectively. In the multivariable analysis, the factors identified as independently related to CRS were ferritin levels at lymphodepletion >673 UI/l (relative risk [RR] 2.5; 95% CI 1.1–5.6; p = 0.025) and having received axi-cel (RR 4.1; 95% CI 1.8–9.5; p = 0.001). For neurotoxicity, the factors were axi-cel (RR 5.4; 95% CI 2.3–12.5; p < 0.001) and a CRP >173 mg/l at lymphodepletion (RR 3.9; 95% CI 1.8–8.3; p < 0.001).

## Discussion

To our knowledge, this is the largest study comparing CAR T-cell therapy with pSOC in RWE. Although the clinical characteristics of the two cohorts differed, multivariate analysis identified an independent influence of treatment cohort on survival, suggesting a significant benefit of CAR-T cell therapy in patients with refractory DLBCL. In addition, as far as we know, we present the first series focused on refractory aBC-NHL patients according to SCHOLAR-1 criteria comparing axi-cel or tisa-cel in the RWE setting. The results of our multivariable analyses indicate better efficacy results for axi-cel compared with tisa-cel, but with increased toxicity.

Regarding the comparison with pSOC, the differences in favor of CAR T-cell therapy in terms OS were also maintained when comparing separately the two products (axi-cel and tisa-cel), although for PFS, statistically significant differences were only identified for axi-cel. In a recent single-center study, Sermer et al. analyzed the use of CAR T-cell therapy versus a historical control of alternative non-CAR T-cell regimens (22), and although they identified a clear benefit in favor of the former, the superiority of CAR T-cell therapy was not as clear in patients with unfavorable prognostic factors. However, in this study, the CAR-T arm had a small number of patients (n = 69) and a separate analysis of refractory patients was not performed. In the same vein, a recently published study compared the results of the SCHOLAR-1 study with the 2-year outcomes of ZUMA-1 (10). All patients in both cohorts fulfilled the SCHOLAR-1 refractoriness criteria, and propensity scoring was used to create balance between ZUMA-1 and SCHOLAR-1 patients. This study showed a notable benefit for axi-cel vs. non-CAR T-cell salvage regimens for patients with refractory DLBCL, with a 73% reduction in the risk of death for this group. In agreement with these results, our study found similar benefits for the CAR-T cohort, but without the selection bias that a clinical trial might incur, given that both cohorts of our study are from the real world. It is noteworthy that the follow-up of both cohorts is dissimilar, with less follow-up for the CAR-T cohort, which could result in a loss of late events in this cohort. However, it should be noted that 92% of the patients had at least 6 months of follow-up, and 68% at least 12 months.

A possible limitation of our study is that the historical cohort received treatment before 2014, mainly regimens based on chemotherapy and rituximab. However, this has been the only treatment available in Spain until very recently, since other options that are currently approved by the EMA, such as polatuzumab vedotin or tafasitamab, were not reimbursed in Spain at the time of designing this study. Further studies are needed to compare CAR-T cell treatment with these new treatment strategies. In addition, due to the high cost of these treatments, it would also be advisable to carry out pharmacoeconomic studies and try to identify the groups of patients that can benefit most from each treatment modality.

Regarding the efficacy and toxicity of CAR T-cell therapy, our results for response rates, PFS, and OS are similar to those of the pivotal trials (13–15) and other previously published RWE studies (17–20), with a best OR rate of 60%, a median PFS of 3.3 months, and a median OS of 11.8 months, with an estimated 12-month OS of 50%. We also observed similar rates of severe CRS (6%) and neurotoxicity (11%) than previously reported in RWE studies but rather less than those identified in the pivotal clinical trials, probably in relation to the anticipation of the use of tocilizumab and steroids. Notably, axi-cel was associated with a significantly higher risk of CRS and especially, severe neurotoxicity. This finding has been previously reported in the pivotal trials (13, 14) as well as in the RWE studies (17–20, 26), and several strategies to prevent neurotoxicity associated with axi-cel, such as prophylaxis with steroids or anakinra (27), are under investigation.

The multivariable analysis of prognostic factors in the CAR-T cell cohort showed, similarly to other studies (17, 18), that unfavorable R-IPI (3–5), ECOG-PS >1, and primary refractory disease had a negative impact on PFS and OS. Although the results in the primary refractory group were inferior, CAR T-cell therapy can lead to long-lasting remissions. Therefore, this subset of patients likely benefits the most from this therapy, if we take into account the poor results achieved with conventional therapy (9). Interestingly, an intermediate-/high-risk HCTCI score (28) was strongly associated with detrimental PFS and OS. This index, widely used in the context of allogeneic transplantation, has been little investigated in the CAR-T therapy setting and could be considered as a useful tool in this context of refractory patients.

When looking at the characteristics of the patients who received axi-cel and tisa-cel in our series, we found significant differences between the two cohorts. Younger patients and more frequently patients with bulky disease pre-apheresis were in the axi-cel group, which was probably related to a bias generated from the physician’s decision when indicating the therapy for this group of refractory patients. This bias could be detrimental to the axi-cel group, given that an association between bulky disease and poorer efficacy results has been identified (12, 17, 26). However, in our study, the efficacy of axi-cel was superior to that of tisa-cel, a finding confirmed in the multivariable analysis, suggesting that axi-cel could be a better option for patients with refractory disease according to SCHOLAR-1 criteria. This superior efficacy of axi-cel was also suggested in a recently published matching-adjusted indirect comparison between ZUMA-1 and JULIET trials, in which the authors concluded that axi-cel may have superior efficacy and greater toxicity than tisa-cel (29). However, this analysis had significant differences form ours because it evaluated clinical trials with different disease refractory definitions, inclusion and exclusion criteria, and study designs. In contrast, our study compared both products used in the commercial setting in a homogeneous population of refractory patients according to SCHOLAR-1 criteria.

The observational nature of our study implies that it is prone to unintentional bias and the effects of confounding variables. Overall and bearing in mind the study’s limitations, the data presented here indicate that the efficacy of CAR T-cell therapy in refractory patients, in terms of PFS and OS, is superior to that of the treatments available in the pre-CAR-T era. In addition, our analyses suggest that axi-cel could be more effective than tisa-cel in refractory patients according to SCHOLAR-1 criteria. Given the limitations of retrospective studies, these results should be confirmed in prospective randomized clinical trials.

## Data Availability Statement

The original contributions presented in the study are included in the article/**Supplementary Material**. Further inquiries can be directed to the corresponding author.

## Ethics Statement

The studies involving human participants were reviewed and approved by the Gregorio Marañón Hospital Ethics Committee. Written informed consent for participation was not required for this study in accordance with the national legislation and the institutional requirements.

## Author Contributions

MB-O, AMG-S, and AG were responsible for the conception and design. MB-O, AG, JR, GI, MT, VO-M, JS, LG-D, RB, AM, PA, RH, HL, J-MS, JD, ASa, CG, LB, SGdV, DG-B, AS, MK, and PB provided the patients and entered the data. MB-O, AMG-S, AG, MK, AS, PB, and AP-M were responsible for the data analysis and interpretation. MB-O, AMG-S, and AG wrote the manuscript. All authors reviewed the paper, gave final approval of the manuscript, and are accountable for all aspects of the work. All authors contributed to the article and approved the submitted version.

## Conflict of Interest

The authors declare that the research was conducted in the absence of any commercial or financial relationships that could be construed as a potential conflict of interest.

## Publisher’s Note

All claims expressed in this article are solely those of the authors and do not necessarily represent those of their affiliated organizations, or those of the publisher, the editors and the reviewers. Any product that may be evaluated in this article, or claim that may be made by its manufacturer, is not guaranteed or endorsed by the publisher.

## Supplementary Material

Supplementary Table 1 Univariable and Multivariable efficacy analysis including all patients, from pSOC and ITT CAR-T cohorts. DLBCL NOS: diffuse large B cell lymphoma not otherwise specified; PML: primary mediastinal lymphoma, HGL DH/TH: high-grade lymphoma doble and triple hit; HGL NOS high-grade lymphoma not otherwise specified. ASCT: autologous stem cell transplantation, R-IPI: reviewed international prognostic index. Multivariable analysis. All variables with a p value less than 0.05 were included in the multivariate analysis. PFS: progression free survival. OS: Overall survival; R-IPI: reviewed international prognostic index, ASCT autologous stem cell transplantation, SOC: standard of care; HR: haze ratio. CI confidence interval.

Supplementary Table 2 Response rates in the CAR-T treated patients, including axi-cel and tisa-cel cohorts. ORR: overall response, CR: complete response, PR: partial response, SD: stable disease. PD: progressive disease, NA not available.
