# Supplementary material for: Best Treatment Option for Patients With Refractory Aggressive B-Cell Lymphoma in the CAR-T Cell Era: Real-World Evidence From GELTAMO/GETH Spanish Groups
Source: Front Immunol. 2022 Jul 12;13:855730. doi: 10.3389/fimmu.2022.855730 (PMC9336530; doi:10.3389/fimmu.2022.855730)
Supplement: Supplementary Table 1 — Univariable and Multivariable efficacy analysis including all patients, from pSOC and ITT CAR-T cohorts. DLBCL NOS: diffuse large B cell lymphoma not otherwise specified; PML: primary mediastinal lymphoma, HGL DH/TH: high-grade lymphoma doble and triple hit; HGL NOS high-grade lymphoma not otherwise specified. ASCT: autologous stem cell transplantation, R-IPI: reviewed international prognostic index. Multivariable analysis. All variables with a p value less than 0.05 were included in the multivariate analysis. PFS: progression free survival. OS: Overall survival; R-IPI: reviewed international prognostic index, ASCT autologous stem cell transplantation, SOC: standard of care; HR: haze ratio. CI confidence interval. [file Table_1.pdf]

Supplementary table 1. Univariable and Multivariable efficacy analysis including all patients, from pSOC and ITT CAR-T cohorts

	Median OS (months)	P	Median PFS (months)	P
**AT DIAGNOSIS**				
**Median age at diagnosis:**		**<0.001**		**0.006**
- **0-60**	15 (NA)		5.6 (3.1-8.1)	
- **>60**	9.3 (6.9-11.7)		4.9 (3.8-6.1)	
**Sex:**		0.17		0.3
- **Male**	11 (8.8-13.2)		4.6 (3.7-5.4)	
- **Female**	14.6 (10.3-18.9)		5.7 (3.7-7.6)	
**Diagnosis:**		0.15		0.031
- **HGL DH/TH**	8.7 (1.2-16.1)		3.3 (1.7-4.9)	
- **HGL NOS**	5.8 (0-14.4)		5 (0-12.5)	
- **DLBCL NOS**	11.2 (9.3-13.1)		5 (4.1-5.8)	
- **Follicular transformed**	NR		13.2 (NA)	
**AA stage:**		0.58		0.88
- **I-II**	13.8 (10.1-17.5)		5.1 (3.1-7.1)	
- **III-IV**	11.4 (9.7-13)		5.1 (3.8-6.3)	
**IPI:**		**0.022**		**0.093**
- **0-2**	16.5 (0-34.4)		5.2 (3.4-7)	
- **3-5**	9.6 (7.6-11.6)		4.7 (4-5.3)	
**PREVIOUS THERAPY**				
**Previous ASCT:**		**<0.001**		**<0.001**
- **Yes**	NR		10.5 (6.3-14.7)	
- **No**	9.3 (7.4-11.2)		4.5 (3.9-5.1)	
**Previous lines:**		0.53		0.34
- **0-2**	12.3 (8.9-15.7)		5.5 (3.6-7.4)	
- **>2**	10 (7.8-12.2)		4.7 (3.7-5.7)	
**STATUS AFTER LAST FAILURE**				
**Therapeutic approach:**		**<0.001**		**0.004**
- **SOC**	8.2 (6.7-9.8)		4.6 (1.5-7.7)	
- **CAR-T**	14.5 (NA)		5.1 (3.5-6.7)	
**Primary refractory:**		0.96		0.59
- **Yes**	11.4 (9.1-13.7)		5.6 (4-7.3)	
- **No**	11.8 (6.9-16.6)		4.8 (3.6-6.1)	

Abbreviations: DLBCL NOS: diffuse large B cell lymphoma no otherwise specified; PML: primary mediastinal lymphoma, HGL DH/TH: high-grade lymphoma doble and triple hit; HGL NOS high-grade lymphoma no otherwise specified. ASCT: autologous stem cell transplantation, R-IPI: reviewed international prognostic index.

Multivariable analysis. All variables with a p value less than 0.05 were included in the multivariate analysis.

	HR	*p* value	95% CI
For PFS			
**Non previous ASCT**	2.13	**<0.001**	1.53-2.97
**Cohort SOC**	1.70	**<0.001**	1.25-2.30
For OS			
**Non previous ASCT**	3.57	**<0.001**	2.26-5.63
**Cohort SOC**	2.24	**<0.001**	1.56-3.21

Abbreviations: PFS: progression free survival. OS: Overall survival; R-IPI: reviewed international prognostic index, ASCT autologous stem cell transplantation, SOC: standard of care; HR: haze ratio. CI confidence interval
