# Supplementary material for: Best Treatment Option for Patients With Refractory Aggressive B-Cell Lymphoma in the CAR-T Cell Era: Real-World Evidence From GELTAMO/GETH Spanish Groups
Source: Front Immunol. 2022 Jul 12;13:855730. doi: 10.3389/fimmu.2022.855730 (PMC9336530; doi:10.3389/fimmu.2022.855730)
Supplement: Supplementary Table 2 — Response rates in the CAR-T treated patients, including axi-cel and tisa-cel cohorts. ORR: overall response, CR: complete response, PR: partial response, SD: stable disease. PD: progressive disease, NA not available. [file Table_2.pdf]

Supplementary table 2. Response rates in the CAR-T treated patients, including axi-cel and tisa-cel cohorts.

	Refractory patients* (N=192)	Axi-cel cohort (N=101)	Tisa-cel cohort (N=91)	*p*
**Response rates at 1 month:**				
- **CR**	55 (29%)	32 (32%)	23 (25%)	0.57
- **PR**	55 (29%)	29 (29%)	26 (29%)	
- **SD**	16 (8%)	9 (9%)	7 (8%)	
- **PD**	41 (21%)	17 (17%)	24 (26%)	
- **NA**	25 (13%)	14 (14%)	11 (12%)	
**Response rates at 3 months:**				
- **CR**	54 (28%)	32 (40%)	22 (34%)	0.40
- **PR**	16 (8%)	10 (12%)	6 (9%)	
- **SD**	9 (5%)	7 (9%)	2 (3%)	
- **PD**	63 (33%)	30 (37%)	33 (52%)	
- **NA**	2 (1%)	1 (1%)	1 (2%)	
**Best response:**				
- **CR**	69 (36%)	42 (42%)	27 (30%)	0.089
- **PR**	46 (24%)	20 (20%)	26 (29%)	
- **SD**	14 (7%)	10 (10%)	4 (4%)	
- **PD**	42 (22%)	17 (17%)	25 (27%)	
- **NA**	21 (11%)	12 (12%)	9 (10%)	
**Ongoing response:**				
- **Ongoing CR**	56 (29%)	32 (32%)	24 (26%)	0.41
- **Ongoing PR**	12 (6%)	6 (6%)	6 (7%)	
- **Ongoing SD**	6 (3%)	5 (5%)	1 (1%)	

Abbreviations: ORR: overall response, CR: complete response, PR: partial response, SD: stable disease. PD: progressive disease, NA not available
