# Supplementary figures and images for: Best Treatment Option for Patients With Refractory Aggressive B-Cell Lymphoma in the CAR-T Cell Era: Real-World Evidence From GELTAMO/GETH Spanish Groups
Source: Front Immunol. 2022 Jul 12;13:855730. doi: 10.3389/fimmu.2022.855730 (PMC9336530; doi:10.3389/fimmu.2022.855730)

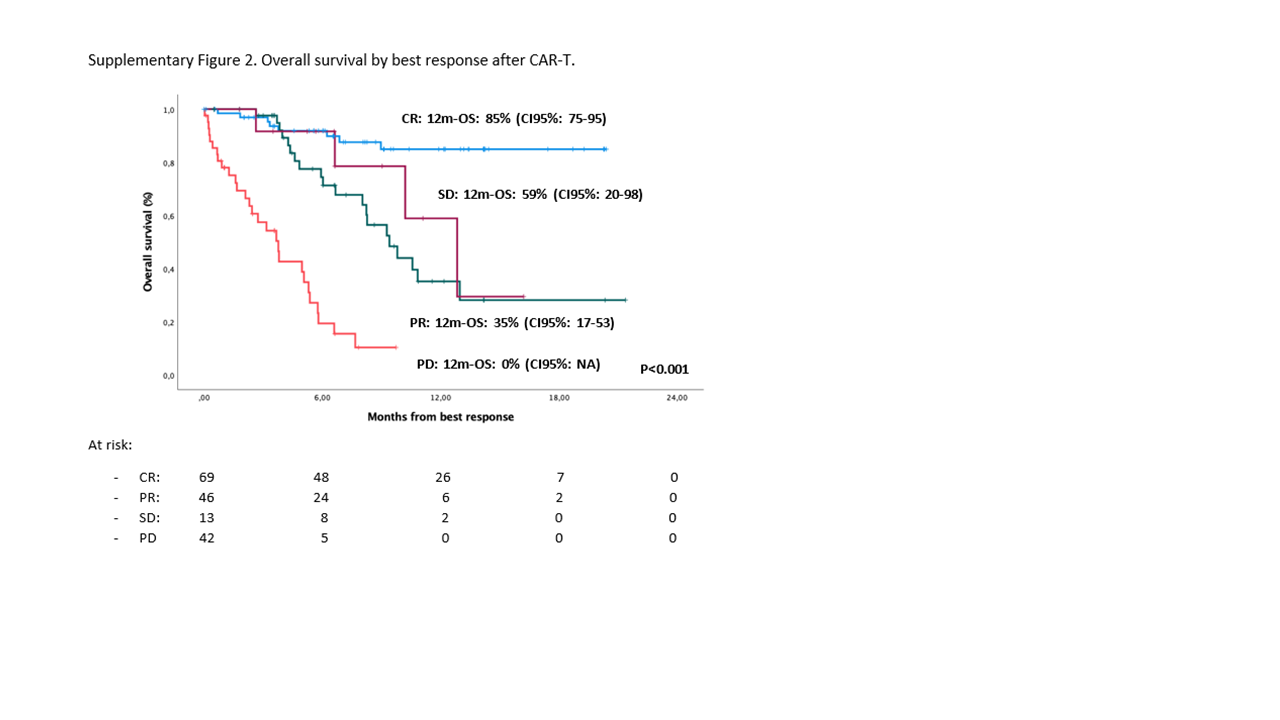

Supplement: Supplementary file 3 [file Image_1.tif]

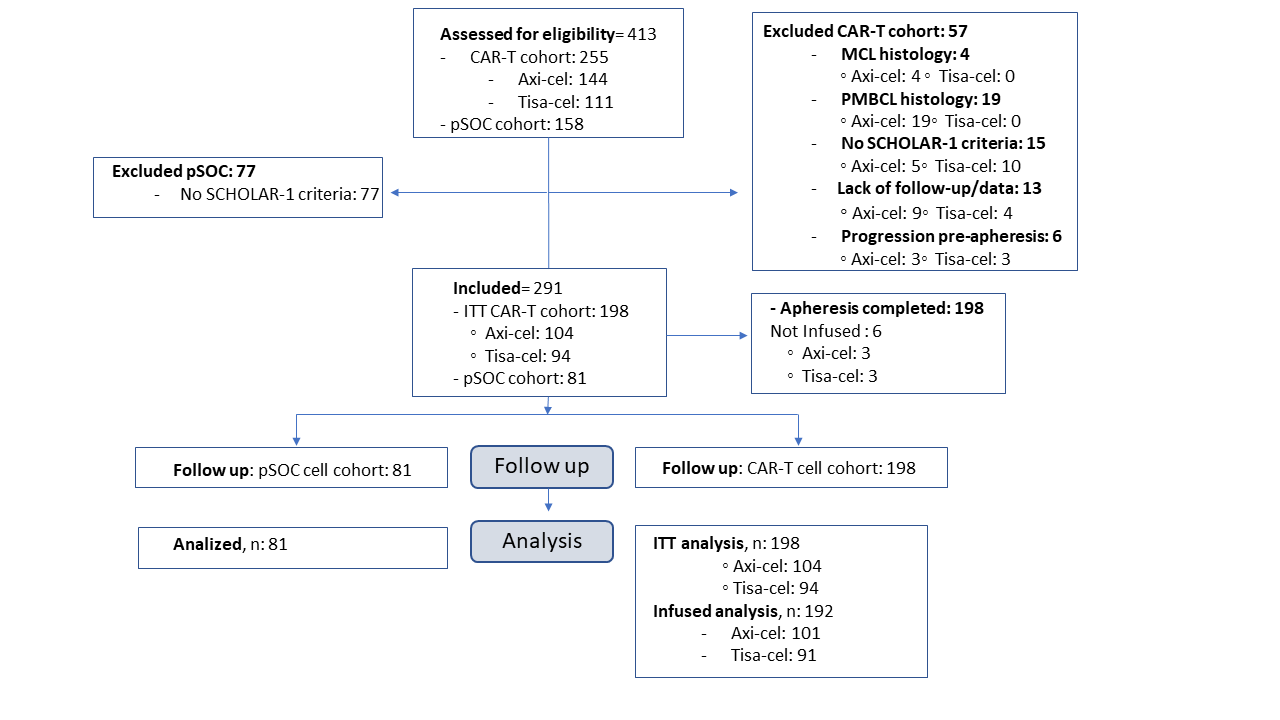

Supplement: Supplementary file 4 [file Image_2.tif]
